# Epigallocatechin-3-gallate opposes HBV-induced incomplete autophagy by enhancing lysosomal acidification, which is unfavorable for HBV replication

**DOI:** 10.1038/cddis.2015.136

**Published:** 2015-05-21

**Authors:** L Zhong, J Hu, W Shu, B Gao, S Xiong

**Affiliations:** 1 Institute for Immunobiology, Department of Immunology, Shanghai Medical College of Fudan University, Shanghai 200032, P.R. China; 2 Jiangsu Key Laboratory of Infection and Immunity, Institutes of Biology and Medical Sciences, Soochow University, Suzhou 215006, P.R. China

## Abstract

Epigallocatechin-3-gallate (EGCG), a major polyphenol in green tea, exhibits diverse beneficial properties, including antiviral activity. Autophagy is a cellular process that is involved in the degradation of long-lived proteins and damaged organelles. Recent evidence indicates that modulation of autophagy is a potential therapeutic strategy for various viral diseases. In the present study, we investigated the effect of EGCG on hepatitis B virus (HBV) replication and the possible involvement of autophagy in this process. Our results showed that HBV induced autophagosome formation, which was required for replication of itself. However, although EGCG efficiently inhibited HBV replication, it enhanced, but not inhibited, autophagosome formation in hepatoma cells. Further study showed that HBV induced an incomplete autophagy, while EGCG, similar to starvation, was able to induce a complete autophagic process, which appeared to be unfavorable for HBV replication. Furthermore, it was found that HBV induced an incomplete autophagy by impairing lysosomal acidification, while it lost this ability in the presence of EGCG. Taken together, these data demonstrated that EGCG treatment opposed HBV-induced incomplete autophagy via enhancing lysosomal acidification, which was unfavorable for HBV replication.

Macroautophagy (hereafter autophagy) is a conserved cellular process through which cytoplasmic materials are sequestered into double-membrane vacuole called autophagosomes and destined for degradation through fusion with lysosomes.^1, 2, 3^ Accumulating evidence indicates that autophagy is involved in diverse pathophysiological processes, including cancer, neurodegenerative disorders, and cardiovascular diseases.^4, 5, 6, 7^ Recent studies show that autophagy has an important role in regulating the replication of many viruses, including dengue virus, coxsackievirus B3 virus (CVB3), hepatitis C virus (HCV), and influenza virus A.^8, 9, 10, 11, 12^ Several investigations also indicate that autophagy has an important role in hepatitis B virus (HBV) replication: autophagy is induced by HBV and is required for HBV replication; however, the underlying mechanisms remains still unclear.^13, 14, 15, 16^

Green tea is the most commonly consumed beverage worldwide. In traditional Chinese medicine, green tea is considered to have beneficial properties for human health, including antitumorigenic, antioxidant, and anti-inflammatory activities.^17, 18, 19^ Epigallocatechin-3-gallate (EGCG) is the most abundant polyphenol in green tea and appears to be the primary active ingredient accounting for the latter's biological effects. In recent years, EGCG is revealed to display inhibitory effect on diverse viruses, such as human immunodeficiency virus type-1, Epstein–Barr virus (EBV), and HCV.^20, 21, 22, 23, 24, 25^ Of interest, EGCG is also found to regulate autophagy formation, although it seems to be cell-type specific.^26, 27, 28, 29, 30^ Given the potential therapeutic effect of EGCG on viral infection and its role in autophagy regulation, we investigated the effect of EGCG on HBV replication and the possible involvement of autophagy in this process.

Here we showed that HBV induced an incomplete autophagy that was required for HBV replication; however, a complete autophagic process induced by EGCG appeared to be unfavorable for HBV replication. Further study showed that HBV hampered the autophagic flux by impairing lysosomal acidification, which could be opposed by the treatment of EGCG.

## Results

### HBV is able to induce autophagosome formation, which is required for replication of itself

**Figure 1:**
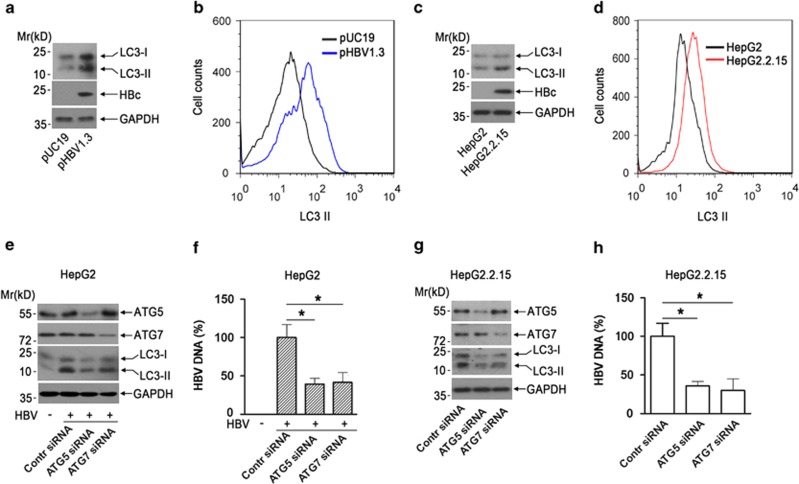
HBV is able to induce autophagosome formation, which is required for replication of itself. (**a**) The effect of HBV transfection on LC3 accumulation in HepG2 cells. HepG2 cells were transfected with empty vector pUC19 or pHBV1.3. Forty-eight hours posttransfection, cells were subjected to western blotting using antibodies against LC3 or HBcAg. The expression of glyceraldehyde 3-phosphate dehydrogenase (GAPDH) was used as a loading control. (**b**) The effect of HBV transfection on autophagosome formation by FACS analysis in HepG2 cells. Cells were transfected with pUC19 or pHBV1.3. Forty-eight hours posttransfection, cells were first washed with phosphate-buffered saline containing 0.05% saponin and then incubated subsequently with anti-LC3 and FITC-labeled second antibody, followed by the FACS analysis. (**c**) Comparison of autophagosome formation in HepG2 with that in HepG2.2.15 cells by western blotting. HepG2 or HepG2.2.15 cells were subjected to western blotting using antibodies against LC3, HBc, and GAPDH as in panel (**a**). (**d**) Comparison of autophagy formation in HepG2 with that in HepG2.2.15 cells by FACS analysis. Cells were subjected to FACS analysis as in panel (**b**). (**e** and **f**) The effect of ATG5 or ATG7 siRNA on autophagome formation in HepG2 cells. HepG2 cells were electro-transfected with ATG5- or ATG7-specific siRNA using Amaxa Nucleofector technology. Forty-eight hours posttransfection, cells were further transfected with pHBV1.3; 48 h later, cells were ubjected to western blotting using antibodies against ATG5, ATG7 or LC3 (**e**), and HBV quantification was determined by quantitative real-time PCR, bars represent means±S.E.M (*n*=3); *P<0.05 (**f**). (**g** and **h**) The effect of ATG5 or ATG7 siRNA on autophagome formation and HBV replication in HepG2.2.15 cells. HepG2.2.15 cells were electro-transfected with ATG5- or ATG7-specific siRNA; 96 h posttransfection, cells were harvested. The expression of ATG5, ATG7 LC3 or GAPDH was determined by western blotting (**g**) and HBV DNA was quantified by real-time PCR, bars represent means±S.E.M (*n*=3); *P<0.05 (**h**)

Accumulating evidence indicates that autophagy has an important role in the regulation of viral replication. So far, the effect of HBV on cell autophagy is still ambiguous. To clarify whether HBV DNA transfection induces autophagy, we transfected empty vector pUC19 and the 1.3 mer HBV DNA (pHBV1.3) into hepatoma HepG2 cells, followed by detecting the autophagosome formation. Western blotting results showed that HBV transfection significantly increased the level of LC3 (microtubule-associated protein light chain 3)-II, a hallmark of autophagy (Figure 1a). We also used fluorescence-activated cell sorting (FACS) analysis to quantify the intracellular LC3-II level as described previously.^31, 32^ As shown in Figure 1b, HBV transfection efficiently increased the saponin-resistant LC3-II^+^ cells. We further compared the autophagosome formation in HepG2 with that in HBV stably transfected HepG2.2.15 cells. Results showed that the autophagosome formation was significantly increased in HepG2.2.15 cells compared with that in HepG2 cells as revealed by western blotting and FACS analysis (Figures 1c and d).

Given the importance of autophagy regulation in viral replication, we examined the role of HBV-induced autophagosome formation in HBV replication by downregulating the expression of ATG5 or ATG7, the key players in autophagosome formation, using small interfering RNA (siRNA) technique in HepG2 cells. Western blotting results showed that ATG5 or ATG7 siRNA transfection efficiently inhibited corresponding protein expression and led to the suppression of autophagosome formation in HepG2 cells (Figure 1e). Of note, results showed that ATG5 or ATG7 siRNA-mediated suppression of autophagosome formation significantly repressed HBV replication (Figure 1f). We also examined the effect of siRNA-mediated downregulation of ATG5 or ATG7 on autophagosome formation and HBV replication in HepG2.2.15 cells. As in Figures 1g and h, blocking autophagosome formation via knockdown of ATG5 or ATG7 significantly repressed HBV replication in HepG2.2.15 cells. Taken together, these data indicated that HBV induced autophagosome formation, which was required for replication of itself.

### EGCG inhibits HBV replication

**Figure 2:**
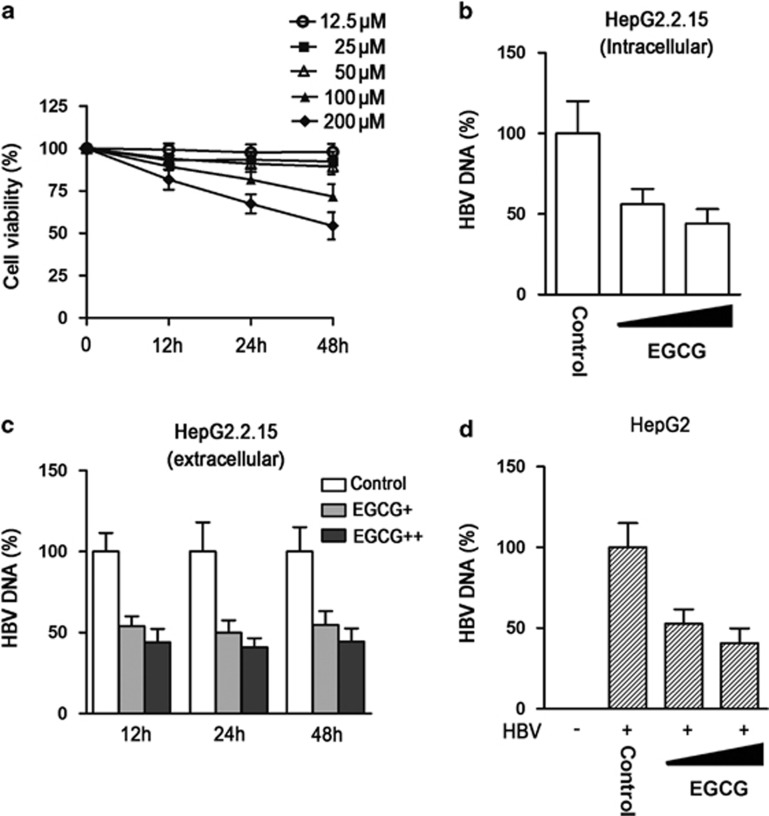
EGCG inhibits HBV replication. (**a**) The effect of EGCG on the viability of HepG2.2.15 cells. HepG2.2.15 cells were treated with increasing amounts of EGCG (12.5, 25, 50, 100, and 200 *μ*M) for the indicated time points (0, 12, 24, and 48 h). The cell viability was determined by CCK-8 method, bars represent means±S.E.M (*n*=3). (**b**) The effect of EGCG on intracellular HBV DNA level in HepG2.2.15 cells. HepG2.2.15 cells were treated with 25 or 50 *μ*M of EGCG for 24 h, and the HBV DNA level in the cell lysates were determined by real-time PCR, bars represent means±S.E.M (*n*=3). **(c)** The effect of EGCG on extracellular HBV DNA level in HepG2.2.15 cells. HepG2.2.15 cells were treated with 25 or 50 *μ*M of EGCG for 24 h, and the HBV DNA level in the cell supernatants were determined by real-time PCR, bars represent means±S.E.M (*n*=3). (**d**) The effect of EGCG on HBV replication in HepG2 cells. HepG2 were transfected with pHBV1.3. Forty-eight hours posttransfection, cells were treated with 25 or 50 *μ*M of EGCG for 24 h. The level of HBV DNA was determined as in panel (**b**)

To investigate the role of EGCG in HBV replication, we first treated HepG2.2.15 cells with different concentrations of EGCG for the indicated times, followed by the detection of cell viability using the Cell Counting Kit-8 (CCK-8) test. The results showed that EGCG with concentration <50 *μ*M had no significant toxic effect on HepG2.2.15 cells (Figure 2a). We then treated HepG2.2.15 cells with 25 or 50 *μ*M of EGCG, followed by the detection of HBV DNA level in cell lysates using quantitative PCR. Results showed that EGCG treatment efficiently downregulated HBV DNA level in HepG2.2.15 cells (Figure 2b). We also collected the culture supernatants of HepG2.2.15 cells and determined the effect of EGCG on extracellular HBV DNA level at various time points. It was found that EGCG treatment dose-dependently suppressed the secretion of HBV DNA by HepG2.2.15 cells; however, we did not observe the time-dependent inhibition of HBV replication by EGCG (Figure 2c). We further tested the effect of EGCG on HBV replication in pHBV1.3-transfected HepG2 cells. Consistent with its effect in HepG2.2.15 cells, EGCG treatment displayed antiviral activity against HBV in a HBV transiently transfected HepG2 cells (Figure 2d).

### EGCG induces, but not inhibits, autophagosome formation in hepatoma cells

**Figure 3:**
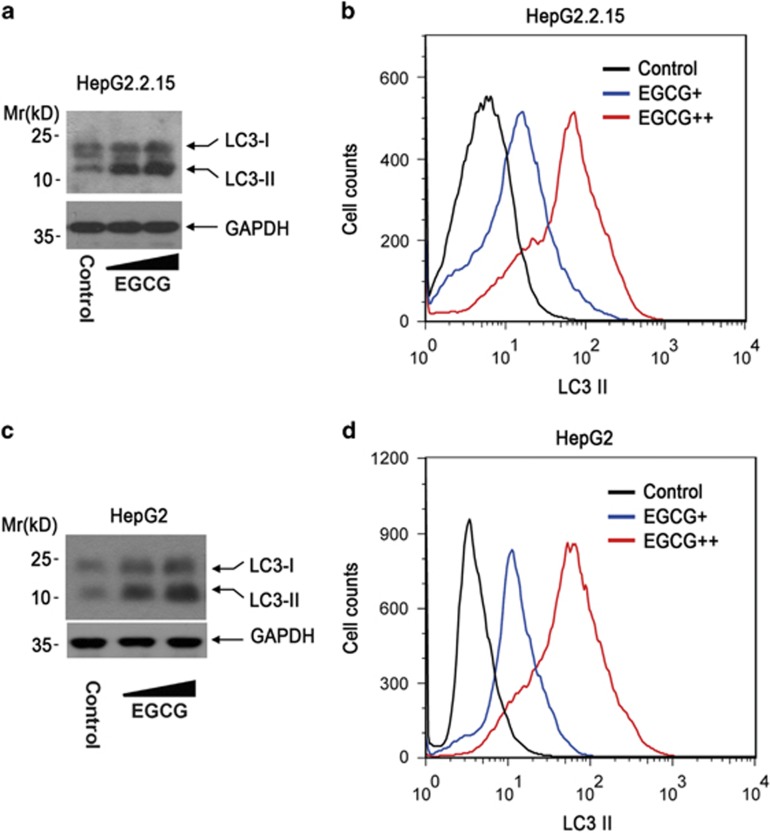
EGCG induces, but not inhibits, autophagosome formation in hepatoma cells. (**a**) The effect of EGCG on autophagosome formation in HepG2.2.15 cells by western blotting. HepG2.2.15 cells were treated with 25 or 50 *μ*M of EGCG for 24 h. Cells were then subjected to western blotting analysis using anti-LC3. The expression of GAPDH (glyceraldehyde 3-phosphate dehydrogenase) was used as a loading control. (**b**) The effect of EGCG on autophagosome formation in HepG2.2.15 cells by FACS analysis. HepG2.2.15 cells were treated with 25 or 50 *μ*M of EGCG for 24 h, and cells were then subjected to FACS analysis as in Figure 1b. (**c** and **d**) The effect of EGCG on autophagosome formation in HepG2 cells. HepG2 cells were treated with 25 or 50 *μ*M of EGCG for 24 h and then subjected to western blotting (**c**) or FACS analysis (**d**)

Given the importance of autophagy in the regulation of HBV replication, we investigated the effect of EGCG on autophagosome formation in hepatoma cells by western blotting and FACS analysis, respectively. Western blotting results showed that EGCG treatment significantly enhanced the LC3-II accumulation in HepG2.2.15 cells (Figure 3a), and FACS analysis also showed that EGCG treatment significantly increased the saponin-resistent LC3-II^+^ cells (Figure 3b). We further determined the effect of EGCG on autophagosome formation in HepG2 cells. Consistent with its effect in HepG2.2.15 cells, EGCG treatment was found to significantly enhance autophagosome formation in HepG2 cells, as evidenced by the results from western blotting (Figure 3c) and FACS analysis (Figure 3d).

### The autophagic process induced by EGCG is different from that by HBV, which appears to be unfavorable for HBV replication

**Figure 4:**
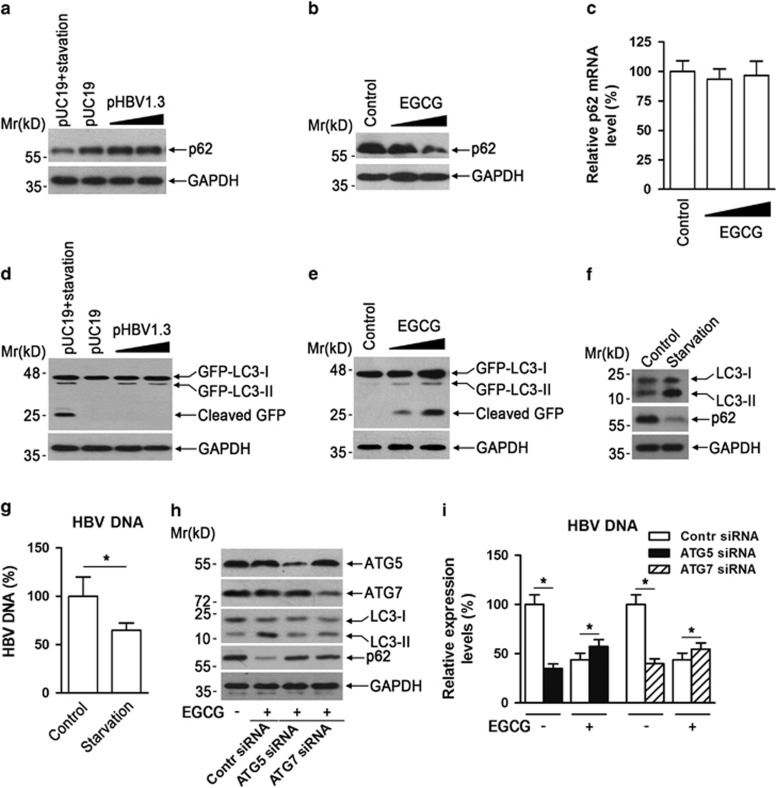
EGCG, but not HBV, enhances autophagic degradation, which appears to be unfavorable for HBV replication. (**a**) The effect of HBV DNA transfection or starvation on p62 degradation in HepG2 cells. Cells were transfected with pUC19 or increasing dose of pHBV1.3 for 48 h. To determine the effect of starvation on p62 degradation, pUC19-transfected HepG2 cells were cultured in Earle's Balanced Salt Solution (EBSS) medium for 3 h. Cells were then subjected to western blotting using anti-p62. The expression of glyceraldehyde 3-phosphate dehydrogenase (GAPDH) was used as a loading control. (**b**) The effect of EGCG on p62 protein level in HepG2 cells. Cells were treated with 25 or 50 *μ*M of EGCG for 24 h and were then subjected to western blotting analysis as in panel (**a**). (**c**) The effect of EGCG on p62 mRNA level in HepG2 cells. Cells were treated as in panel (**b**), and p62 mRNA expression level was determined by quantitative reverse transcriptase-PCR, bars represent means±S.E.M (*n*=3). (**d**) The effect of HBV DNA transfection or starvation on GFP-LC3 processing. HepG2 cells stably expressing GFP-LC3 were transfected with pUC19 or increasing dose of pHBV1.3 for 48 h. To determine the effect of starvation on GFP-LC3 processing, pUC19-transfected cells were cultured in EBSS medium for 3 h. Cells were then subjected to western blotting with anti-GFP. The expression of GAPDH was used as a loading control. (**e**) The effect of EGCG on GFP-LC3 processing. GFP-LC3 stably transfected HepG2 were treated with 25 or 50 *μ*M of EGCG for 24 h, and cells were then subjected to western blotting as in panel (**d**). (**f**) The effect of starvation on autophagosome formation and p62 degradation. HepG2.2.15 cells were cultured in EBSS medium for 3 h and were then subjected to western blotting using antibodies against LC3 and p62. The expression of GAPDH was used as a loading control. (**g**) The effect of starvation on HBV replication. HepG2.2.15 cells. were treated as in panel (**f**) and subjected to HBV DNA quantification using real-time PCR, bars represent means±S.E.M (*n*=3); *P<0.05. (**h**) The effect of ATG5 or ATG7 siRNA on EGCG-induced autophagy. HepG2.2.15 cells were electro-transfected with ATG5- or ATG7-specific siRNA. Seventy-two hours later, cells were subjected to the EGCG treatment for another 24 h. The expression of ATG5, ATG7, LC3, or p62 was determined by western blotting. GAPDH was used as a loading control. (**i**) The effect of ATG5 or ATG7 siRNA on EGCG-mediated inhibition of HBV replication. HepG2.2.15 cells were treated as in panel (**h**) and subjected to HBV DNA quantification using real-time PCR, bars represent means±S.E.M (*n*=6). *P<0.05

As the above data demonstrated that HBV-induced autophagy was required for replication of itself, while EGCG inhibited HBV replication even though it strongly induced autophagosome formation, we wanted to know the reason for this discrepancy. Reports indicate that a normal autophagy flux is important for the regulation of diverse phsiopathological processes.^33, 34^ We thus compared the effect of HBV or EGCG on the protein level of SQSTM1/p62 (p62), which is incorporated into autophagosomes and degraded along with other substrates by lysosomal hydrolyses.^2, 3^ As shown in Figure 4a, HBV DNA transfection failed to cause the degradation of p62, while starvation, a canonical autophagy inducer, significantly downregulated p62 protein level in HepG2 cells, as expected. We further investigated the effect of EGCG on p62 degradation in HepG2 cells. It was found that EGCG treatment dose-dependently decreased p62 protein level (Figure 4b). To exclude the possibility that EGCG-mediated downregulation of p62 protein level was due to its effect on p62 transcription, we examined the effect of EGCG on p62 mRNA expression level by quantitative RT-PCR. Results showed that EGCG treatment did not affect p62 mRNA level significantly (Figure 4c). It is reported that, in cells expressing GFP-LC3, the GFP-LC3 processing is also useful to monitor autophagic degradation.^31, 35^ We thus also investigated the effect of HBV or EGCG on GFP-LC3 processing in HepG2 cells. Consistent with the degradation of p62, treatment with EGCG or starvation, but not with HBV, led to the cleavage of GFP-LC3 (Figures 4d and e).

Above data showed that the HBV-induced incomplete autophagy was required for HBV replication, whereas EGCG induced a complete autophagic process and inhibited HBV replication We therefore also tested the effect of starvation on HBV replication. It was found that starvation treatment significantly increased LC3-II level and led to the downregulation of p62 protein level, as expected (Figure 4f); however, it also failed to enhance HBV replication, and to some extent, even inhibited HBV replication in HepG2.2.15 cells. (Figure 4g). We further evaluated the EGCG-induced autophagy on HBV replication by knockdown of ATG5 or ATG7 in HepG2.2.15 cells. The results showed that ATG5 or ATG7 knockdown significantly downregulated the EGCG-induced LC3-II level and attenuated the EGCG-mediated p62 degradation in HepG2.2.15 cells (Figure 4h), indicating ATG5 or ATG7 knockdown suppressed EGCG-induced complete autophagic process. Further, it was found that although ATG5 or ATG7 knockdown significantly suppressed HBV replication in the absence of EGCG, it did not have additive inhibitory effects on HBV replication with EGCG but could reverse EGCG-mediated inhibition of HBV replication to a moderate extent (Figure 4i). Also, in consideration of the limited siRNA transfection efficency, these data indicated that the EGCG-induced complete autophagic process was unfavorable for HBV replication, although it might not be responsible for all the EGCG-mediated anti-HBV activity.

### EGCG opposes HBV-induced incomplete autophagy

**Figure 5:**
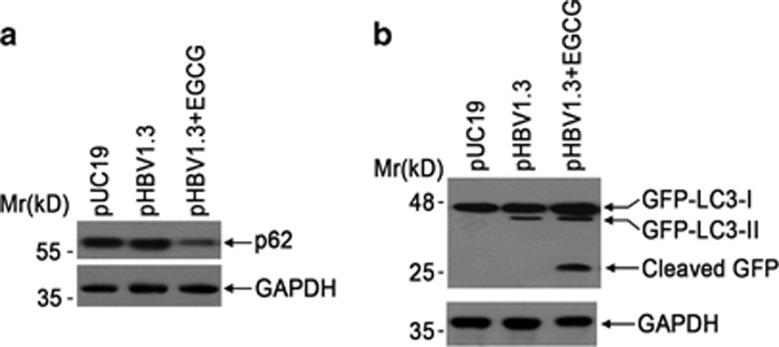
EGCG opposes HBV-induced incomplete autophagy. (**a**) The effect of EGCG on p62 protein expression level. HepG2 cells were transfected with pUC19 or pHBV1.3. Forty-eight hours later, pHBV1.3-transfected cells were treated with or without 50 *μ*M of EGCG for 24 h, and then subjected to western blotting using anti-p62 antibody. The expression of glyceraldehyde 3-phosphate dehydrogenase (GAPDH) was used as a loading control. (**b**) The effect of EGCG treatment on GFP-LC3 processing. GFP-LC3-transfected HepG2 cells were transfected with pUC19 or pHBV1.3; 48 h later, HBV-transfected cells were was treated with or without 50 *μ*M of EGCG for 24 h, followed by the western blotting using anti-GFP. The expression of GAPDH was used as a loading control

As above data showed that HBV induced an incomplete autophagy, whereas EGCG induced a complete one, we wanted to know the effect of EGCG on HBV-induced autophagy. We transfected pHBV1.3 into HepG2 cells, followed by the treatment with or without EGCG for 24 h. As shown in Figure 5a, although HBV transfection failed to result in the p62 degradation, EGCG treatment significantly downregulated p62 protein level in HBV-transfected cells. We also determined the effect of HBV on GFP-LC3 cleavage in the presence of EGCG. Consistent with p62 degradation, the cleavage of GFP-LC3 was clearly observed in pHBV1.3-transfected cells after treatment with EGCG (Figure 5b), further indicating that EGCG opposed HBV-induced incomplete autophagy.

### EGCG opposes HBV-induced incomplete autophagy by increasing lysosomal acidification

**Figure 6:**
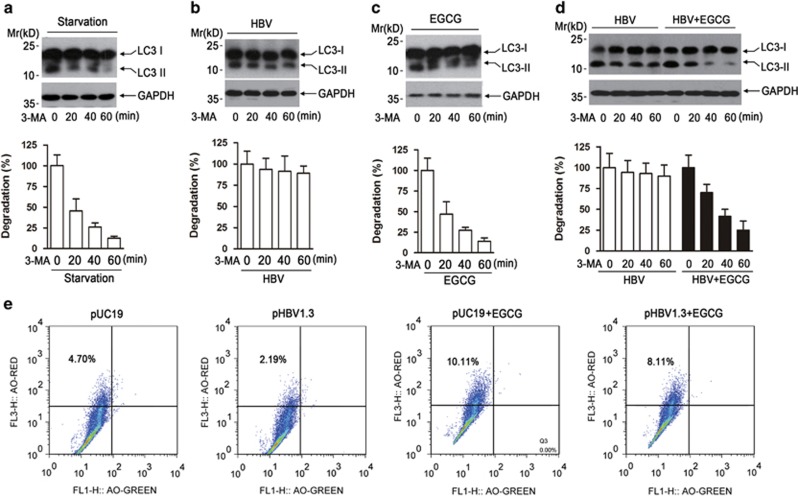
EGCG opposes HBV-induced incomplete autophagy by increasing lysosomal acidification. (**a**) The effect of starvation on the degradation of formed autophagesome. HepG2 cells were cultured in Earle's Balanced Salt Solution medium for 3 h, and the pI3KC3 inhibitor 3-MA (10 mM) was added to stop the formation of new autophagosome. At the indicated time points (0, 20, 40, 60 min) after 3-MA addition, western blotting was used to determine the level of LC3-II. The expression of glyceraldehyde 3-phosphate dehydrogenase (GAPDH) was used as a loading control. Lower panel, the immunoblots from three independent experiments were scanned and subjected to densitometric analysis. The density value from starved cells without 3-MA treatment was set as 100%, bars represent means±S.E.M (*n*=3). (**b**) The effect of HBV transfection on the degradation of formed autophagesome. HepG2 cells were transfected with pHBV1.3 for 48 h. The degradation of formed autophagosome was examined as in panel (**a**). (**c**) The effect of EGCG treatment on the degradation of formed autophagesome. HepG2 cells were treated with EGCG for 24 h, and the degradation of formed autophagosome was examined as in panel (**a**). (**d**) The effect of HBV transfection on the degradation of formed autophagesome in the presence of EGCG. HepG2 cells were transfected with pHBV1.3. Forty-eight hours posttransfection, cells were then treated or untreated with 50 *μ*M of EGCG for another 24 h. The degradation of formed autophagesome was determined as in panel (**a**). (**e**) The effect of HBV on the lysosomal acidification in the presence or absence of EGCG. HepG2 cells were transfected with pUC19 or pHBV1.3 for 48 h, followed by treatment with or without 50 *μ*M of EGCG for 24 h. Cells were then stained with AO for 15 min and subjected to FACS analysis

To decipher the mechanism whereby EGCG opposed HBV-induced incomplete autophagy, we determined the effect of HBV or EGCG on the degradation of formed autophagosome using a pulse-chase assay as described by Liu *et al.*^35^ The results revealed that, once preventing the synthesis of new autophagosome with the pI3KC3 inhibitor 3-methyladenine (3-MA), the starvation-induced autophagosome was degraded rapidly (Figure 6a), while HBV was found to hamper the degradation of formed autophagosome (Figure 6b), indicating that HBV impaired the degradative capacity of lysosomes. Similar to starvation treatment, EGCG was also found to enhance the degradation of formed autophagosome (Figure 6c). We further determined the effect of HBV on the degradation of formed autophagosome in the presence of EGCG. It was found that HBV lost its ability to inhibit the degradation of formed autophagosome after EGCG treatment (Figure 6d).

It is now known that the acidification is crucial for the activation of lysosomal hydrolases and maintaining acidity is an important marker of normal lysosomal function.^1, 2, 3, 36^ We thus tested the effect of HBV on lysosomal acidification by acridine orange (AO) staining. Results showed that, although EGCG expectedly enhanced lysosomal acidification, HBV transfection failed to do so and even impaired this process in comparison to empty vector-transfected cells (Figure 6e). Of note, in the presence of EGCG, the lysosomal acidification was strongly enhanced in HBV-transfected cells (Figure 6e).

## Discussion

In recent years, autophagy has been revealed to have a crucial role in the replication of many viruses. Manipulation of autophagy is therefore regarded to be a potential therapeutic strategy for viral infection. In the present investigation, our data showed that HBV-induced autophagosome formation was required for replication of itself, consistent with previous investigations.^14, 15, 16^ Besides HBV, autophagy has also been exploited to promote the replication of several RNA and DNA viruses, such as HCV, CVB3, and Kaposi's sarcoma-associated herpesvirus,^37, 38, 39^ although the precise underlying mechanisms have not been fully delineated. Several mechanisms have been suggested to contribute to the proviral activities of autophagy. Some viruses may subvert autophagosome to use as scaffolds supporting their assembly and replication. As a pro-survival stragtegy, autophagy may help keep alive those virally infected cells, allowing persistent viral replication. As autophagy is also a pathway to degradation, viruses have developed strategies to block autophagosome maturation to escape lysosomal degradation.^3, 12, 40, 41, 42^ Recent evidence indicates that EBV blocks the autophagic flux and appropriates the autophagic vesicles for viral transportation into the cell cytoplasm and avoid viral degradation into the lysosomes.^43^

EGCG is the main constituent of tea, which has been demonstrated to possess diverse beneficial effects to human health, including antiviral activity. Our data showed that EGCG treatment efficiently inhibited HBV replication. Similar to our results, other groups also revealed that EGCG possessed antiviral activity against HBV.^44, 45^ Given the importance of autophagy in regulating HBV replication, we investigated the effect of EGCG on autophagosome formation in hepatoma cells. Our results showed that EGCG strongly induced autophagosome formation in hepatoma HepG2.2.15 and HepG2 cells. We also tested the effect of EGCG on autophagosome formation in another hepatoma cell line, Huh7, and had obtained similar results as that in HepG2.2.15 and HepG2 cells (data not shown). Consistent with our results, Zhou *et al*^28^ also reported that EGCG enhanced autophagy induction in hepatocytes. However, there is report that EGCG showed significant inhibitory effect on autophagy signaling in hepatoma Hep3B cells.^29^ The reason for this discrepancy is unclear and may be due to the use of different hepatic cell lines.

As HBV-induced autophagy was required for HBV replication, whereas EGCG-induced autophagy did not facilitate HBV replication, we thus liked to know whether there existed difference between HBV- and EGCG-induced autophagy. p62 protein is incorporated into autophagosomes and degraded along with other substrates by lysosomal hydrolyses and widely used to monitor autophagic flux. We found that HBV genomic DNA transfection failed to cause the degradation of p62, consistent with previous investigation,^14, 46^ while EGCG treatment dose-dependently decreased p62 protein level. We further investigated the effect of HBV transfection or EGCG on GFP-LC3 processing in HepG2 cells. Consistent with the degradation of p62, treatment with EGCG or starvation, but not with HBV, led to the cleavage of GFP-LC3, indicating that HBV induced an incomplete autophagy, while EGCG, similar to stavation, induced a complete autophagic process. We further investigated the effect of starvation, a canonical autophagy inducer, on HBV replication and found that starvation treatment significantly enhanced the formation of autopagosome and p62 degradation, as expected; however, it also failed to enhance HBV replication, and to some extent, even inhibited HBV replication. We have also investigated the effect of EGCG-induced complete autophagic process on HBV replication by knockdown of ATG5 or ATG7 using siRNA technique. It was found that knockdown of ATG5 or ATG7 significantly inhibited EGCG-induced complete autophagic process, and it did not have additive inhibitory effects with EGCG on HBV replication but could moderately reverse EGCG-mediated inhibition of HBV replication, indicating that the EGCG-induced complete autophagic process was unfavorable for HBV replication. It was also found that the inhibitory effect on HBV replication by EGCG was stronger than that by starvation; we therefore did not exclude the possibility that EGCG, as a versatile antiviral molecule, might inhibit HBV replication via other mechanisms.

Our data further showed that, in the presence of EGCG, the p62 degradation and GFP-LC3 processing was significantly enhanced in HBV-transfected cells, indicating that EGCG opposed HBV-induced incomplete process. To further decipher the underlying mechanism, we determined the effect of HBV or EGCG on the degradation of formed autophagosome using a pulse-chase assay. Our results revealed that, although EGCG-induced autophagosome was degraded rapidly in the presence of pI3KC inhibitor 3-MA, HBV transfection hampered the degradation of formed autophagosome, indicating that HBV impaired the degradative capacity of lysosomes. Consistent with our results, Liu *et al*^35^ reported that HBV X protein possesses the ability to inhibit lysosmal degradation. As it is well established that the lysosomal acidification is crucial for the degradation of engulfed materials and is an important marker of functional lysosome,^1, 2, 3, 46^ we thus tested the effect of HBV on lysosomal acidification by AO staining. It was found that HBV transfection impaired lysosomal acidification; however, this process could be opposed by EGCG treatment.

In summary, our data showed that HBV induced an incomplete autophagy in hepatoma cells. Although autophagosome formation was required for HBV replication, EGCG- or starvation-induced complete autophagic process appeared to be unfavorable for HBV replication. Further mechanistic study showed that HBV hampered the degradative activity of lysosome by impairing lysosomal acidification, which was opposed by EGCG. These data may help to clarify the role of autphagy in viral replication and control viral infection by autophagic manipulation.

## Materials and Methods

### Cell culture, treatment, and transfection

HepG2 cells and HepG2.2.15 cells, which constitutively replicates HBV, were maintained at 37 °C in DMEM supplemented with 10% FBS and antibiotics in a 5% CO_2_ atmosphere. Where indicated, cells were treated with EGCG (Sigma, St. Louis, MO, USA). HBV DNA transient transfection was performed by transfecting empty vector pUC19 and the 1.3mer HBV DNA (pHBV1.3) into HepG2 cells using lipofectamine 2000 reagent (Invitrogen, Carlsbad, CA, USA). For siRNA transfection in HepG2 or HepG2.2.15 cells, cells were trypsinized and mixed with 100 nM of ATG5, ATG7, or control siRNA (GenePharma, Shanghai China) and then subjected to electrotransfection using Amaxa Nucleofector technology (Amaxa GmbH, Köln, Germany).

### Western blotting

Western blotting was performed as described previously.^47, 48, 49^ Antibodies against LC3 and p62 were from MBL International (Woburn, MA, USA). Antibodies against ATG5 or ATG7 were obtained from Cell Signaling Technology (Beverly, MA, USA). Anti-HBcAg was from DAKO (Glostrup, Denmark).

### HBV DNA analysis

The intracellular HBV core particles were prepared as previously described.^49^ The cytoplasmic HBV DNA was extracted by a Viral Genome Purification Kit (Cwbiotech, Beijing, China), and the quantification of HBV DNA was determined by real-time PCR using an HBV Diagnostic Kit (Kehua Biotech, Shanghai, China) according to the manufacturer's instructions.^50^

### Determination of saponin-resistant LC3-II^+^ positive cells

Saponin-resistant LC3-II+ positive cells were determined by FACS analysis as described previously.^30, 31^ Briefly, cells were harvested with trypsin, washed with PBS, and then permeabilized with 0.05% saponin in PBS. Cells were then incubated with mouse anti-LC3 primary antibody for 30 min, rinsed with PBS, incubated with FITC-labeled anti-mouse second antibody (Bioworld, St. Louis Park, MN, USA) for 20 min, rinsed twice with PBS, and resuspended in 0.5 ml of PBS. Flow cytometry was carried out on a FACS Calibur (BD Biosciences, San Jose, CA, USA) and data were analyzed by the FlowJo software (Tree Star, Ashland, OR, USA).

### Effect of EGCG on cell viability

HepG2.2.15 cells were plated in 96-well culture plates at 2000 cells/well in a final volume of 100 *μ*l of complete medium and were allowed to attach for 2 days. The cells were then treated with varying concentrations of EGCG for 12, 24, and 48 h. After the completion of each treatment, 20 *μ*l of CCK-8 solution was added to each well. The cells were incubated for 2 h, after which absorbance was measured at 450 nm. Values were expressed as a percentage relative to those obtained in the control groups.

### Quantitative real-time RT-PCR

Extraction of total RNA reverse transcription was performed as previously described.^47, 48, 49^ cDNA was subjected to quantitative RT-PCR with the following primers: p62, forward, 5′-CGGGTGGGAATGTTGAGG-3′ and reverse, 5′-TGGCGGGAGATGTGGGTAC-3′ and GAPDH, forward, 5′-ATCCCATCACCATCTTCCAG-3′ and reverse, 5′-GAGTCCTTCCACGATACCAA-3′. The expression level of p62 was calculated following normalization to GAPDH level by the comparative ΔΔ threshold cycle method.

### Pulse-chase assay to measure the degradation of formed autophagosomes

The pulse-chase assay to measure the degradation of formed autophagosomes was adapted from the method of Liu *et al.*^46^ Briefly, HepG2 cells were cultured in EBSS medium for 3 h or transfected with pHBV1.3 for 48 h or treated with EGCG for 24 h. Where indicated, HBV-transfected cells were treated with EGCG for another 24 h. Cells were then treated with 10 mM 3-MA to stop the formation of new autophagosome. At the indicated time points (0, 20, 40, 60 min) after 3-MA treatment, cells were collected and subjected to western blotting analysis using anti-LC3 or GAPDH.

### AO staining

AO staining was used to evaluate the lysosomal acidification as described before.^41^ Briefly, cells were cultured with 0.5 *μ*g/ml AO (Sigma) for 15 min at 37 °C. After washing with PBS for three times to remove excess AO, the cells were dislodged with trypsin. Cells were then collected by centrifugation and resuspended in 0.5 ml of PBS for analysis within 2 h using a FACS Calibur, and data were analyzed with the Flowjo software.

### Statistical analysis

Data were presented as means±S.E.M. Student's *t*-test was applied to compare between groups; a *P* value <0.05 was considered to be statistically significant.

## Abbreviations

EGCG: epigallocatechin-3-gallate
HBV: hepatitis B virus
HCV: hepatitis C virus
CVB3: coxsackievirus B3 virus
EBV: Epstein–Barr virus
LC3: microtubule-associated protein light chain 3
FACS: fluorescence-activated cell sorting
siRNA: small interfering RNA
3-MA: 3-methyladenine
AO: acridine orange
